# Cancer cachexia: biomarkers and the influence of age

**DOI:** 10.1002/1878-0261.13590

**Published:** 2024-02-27

**Authors:** Julia Geppert, Maria Rohm

**Affiliations:** ^1^ Institute for Diabetes and Cancer Helmholtz Munich Neuherberg Germany; ^2^ Joint Heidelberg‐IDC Translational Diabetes Program, Inner Medicine 1 Heidelberg University Hospital Germany; ^3^ German Center for Diabetes Research (DZD) Neuherberg Germany

**Keywords:** ageing, biomarker, cachexia, cancer, inflammation, metabolism

## Abstract

Cancer cachexia (Ccx) is a complex metabolic condition characterized by pronounced muscle and fat wasting, systemic inflammation, weakness and fatigue. Up to 30% of cancer patients succumb directly to Ccx, yet therapies that effectively address this perturbed metabolic state are rare. In recent decades, several characteristics of Ccx have been established in mice and humans, of which we here highlight adipose tissue dysfunction, muscle wasting and systemic inflammation, as they are directly linked to biomarker discovery. To counteract cachexia pathogenesis as early as possible and mitigate its detrimental impact on anti‐cancer treatments, identification and validation of clinically endorsed biomarkers assume paramount importance. Ageing was recently shown to affect both the validity of Ccx biomarkers and Ccx development, but the underlying mechanisms are still unknown. Thus, unravelling the intricate interplay between ageing and Ccx can help to counteract Ccx pathogenesis and tailor diagnostic and treatment strategies to individual needs.

## Graphical abstract

Cancer cachexia is a wasting disease associated with many cancers that leads to alterations in systemic metabolism, a loss of muscle and adipose tissue, and inflammation. In the current review, key mechanisms of cachexia are introduced, and how they relate to current knowledge on cachexia biomarkers. The interplay between ageing and cancer cachexia regarding potential links and biomarkers is discussed.

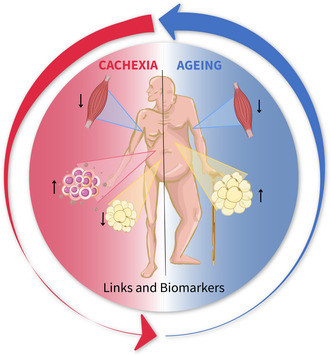

**Abbreviations**
AA: amino acid
ACC: acetyl‐CoA carboxylase
AKT: protein kinase B
AMP: adenosine monophosphate
AMPK: AMP‐activated protein kinase
ATGL: adipose triglyceride lipase
ATP: adenosine triphosphate
BAX: B cell leukaemia/lymphoma 2 (BCL2)‐ associated X protein
BMI: body mass index
BMP: bone morphogenetic protein
Bnip3: BCL2 interacting protein 3
C1r: complement C1r subcomponent
C26: colon 26
C7: complement component 7
CASCO: cachexia score
Ccx: cancer cachexia
CEBPα: CCAAT enhancer binding protein alpha
CER: ceramide
CRP: C‐reactive protein
CXI: cachexia index
DGAT: diacylglycerol O‐acyltransferase
ESPEN: European Society of Clinical Nutrition and Metabolism
F2: coagulation factor II
FAS: fatty acid synthase
FFA: free fatty acid
FoxO: forkhead box O
GDF15: growth differentiation factor 15
GNRI: Geriatric Nutritional Risk Index
HCER: hexosyl‐ceramide
HSL: hormone sensitive lipase
ICV: intracerebroventricular
IFNAR1: interferon alpha and beta receptor subunit 1
IFNγ: interferon gamma
IL: interleukin
LC3: microtubule‐associated protein 1A/1B‐light chain 3
LLC: Lewis lung cancer
LPC: lysophosphatidylcholine
LPS: lipopolysaccharide
LYVE1: lymphatic vessel endothelial hyaluronan receptor 1
MAFbx: muscle atrophy F box, also termed atrogin 1
MCP‐1: monocyte chemoattractant protein 1
mTOR: mammalian target of rapamycin
MuRF1: muscle RING finger 1
NF‐κB: nuclear factor kappa‐light‐chain‐enhancer of activated B cells
NLR: neutrophil‐to‐lymphocyte ratio
PLA2G7: phospholipase A2 group VII
PPARγ: peroxisome proliferator activated receptor gamma
ROS: reactive oxygen species
SM: sphingomyelin
STAT3: signal transducer and activator of transcription 3
TGFβ: transforming growth factor beta
TIMP1: tissue inhibitor of metalloproteinases‐1
TLC: tissue inhibitor of metalloproteinases‐1/liver/cachexia score
TNFα: tumour necrosis factor alpha
UCP: uncoupling protein
UPS: ubiquitin proteasome system
ZAG: zinc‐α2‐glycoprotein

## 1 Introduction

Cancer cells reprogram their metabolism to meet their energetic and cellular demands, ultimately aiming to evolve and sustain favourable or—for the host—malignant properties. In addition to altering their cellular metabolism, specific cancer types also induce systemic reprogramming of the host's energy metabolism, leading to alterations in glucose [1], lipid [2, 3] and protein turnover [4]. These metabolic imbalances are fostered by tumour‐secreted and tumour‐induced host‐derived factors promoting a wasting syndrome termed cancer cachexia (Ccx) [5, 6]. Ccx is a multifactorial disease associated with many cancer types, with an occurrence of up to 87% in gastric and pancreatic cancers [7]. Cancer‐associated weight loss is linked to poor overall survival, reduced treatment success, and is estimated to cause 20–30% of cancer‐related deaths [8]. Hence, to date, counteracting cachexia represents a critical unmet medical need. Being a multi‐organ disease [9], inter‐organ communication by circulating factors contributes to pathogenesis and simultaneously may aid in the identification of affected patients. In addition to finding universal Ccx biomarkers, recent studies have highlighted the importance of variable metabolic predisposition in disease detection and progression, taking into account for instance sex [10, 11], metabolic state [12] and age [13]. Personalized therapeutic approaches should be prioritized based on recent advances in cancer research [14], which have shown the effectiveness of individualized strategies. However, considering that cancer is primarily a disease of higher age, there is a lack of research on the impact of age on cachexia development, highlighting the need for increased focus on this topic in future studies.

**Fig. 1:**
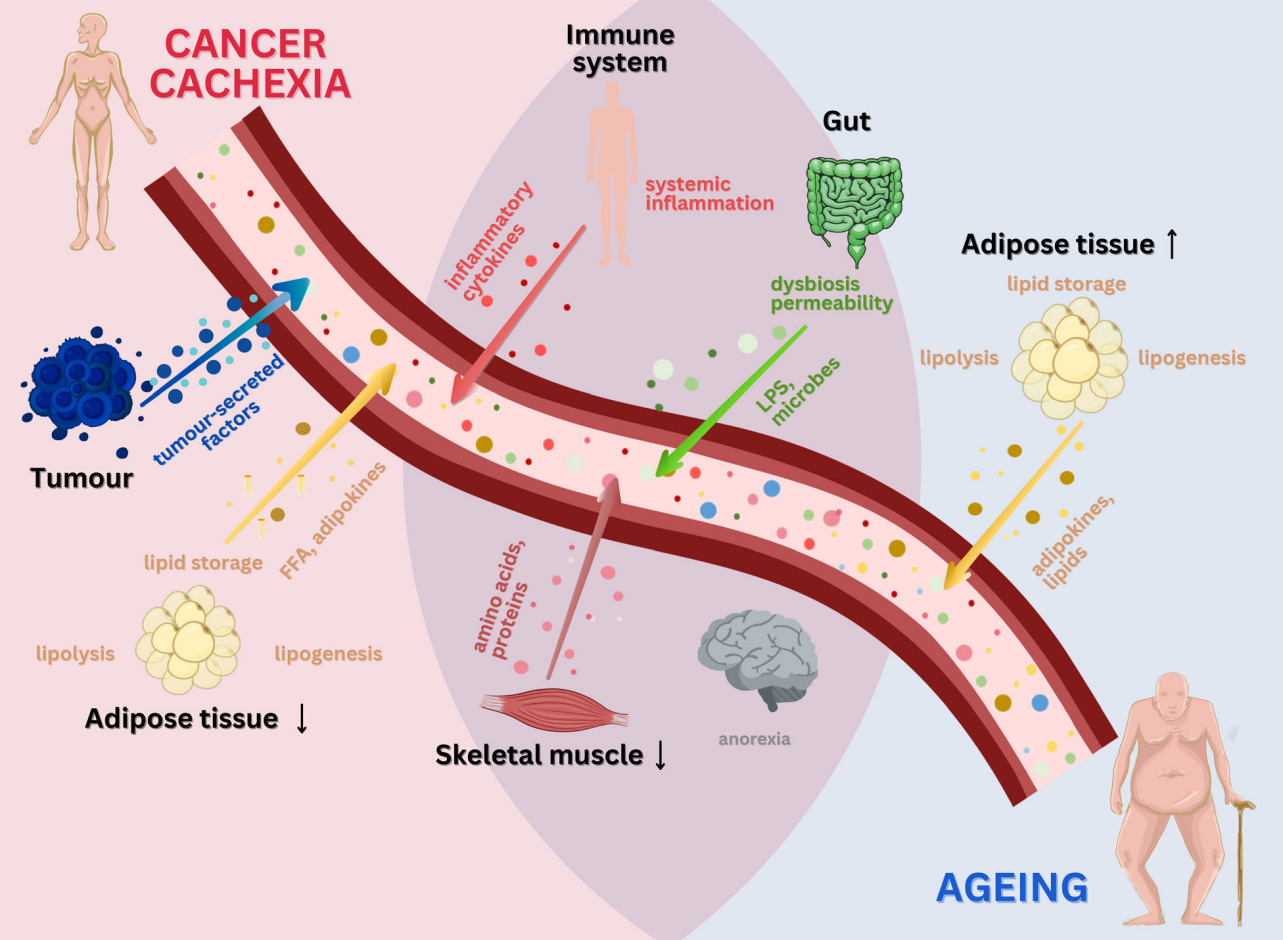
The interplay of cancer cachexia and ageing with a focus on biomarkers in disease progression. Factors released from tumour and different diseased tissues contribute to the pathogenesis of cachexia and metabolic dysfunction upon ageing, and can be used as biomarkers. The affected processes partially overlap. Abbreviations shown in abbreviation list.

The current review discusses typical molecular features of cachexia, recent advances in biomarker discovery related to these features and the first steps towards a more personalized approach to diagnosis, with a special focus on patient age (Fig. 1).

## 2 Cancer cachexia characteristics in brief

Patients with cachexia suffer from marked and unstoppable wasting of adipose tissue and skeletal muscle, overall leading to severe loss of bodyweight [15, 16, 17]. While anorexia contributes to cachexia, metabolic dysregulation dominates the syndrome, as nutritional approaches cannot fully restore energy homeostasis [17]. Altered metabolism and signalling in Ccx affect multiple organs including liver, heart, brain, the immune system, adipose tissue and skeletal muscle [9]. The disease evolves progressively with continuous adaptations in key metabolic processes as recently reviewed [18], thus enabling to deduct biomarker strategies based on the stage of cachexia‐specific metabolic alterations.

**Fig. 2:**
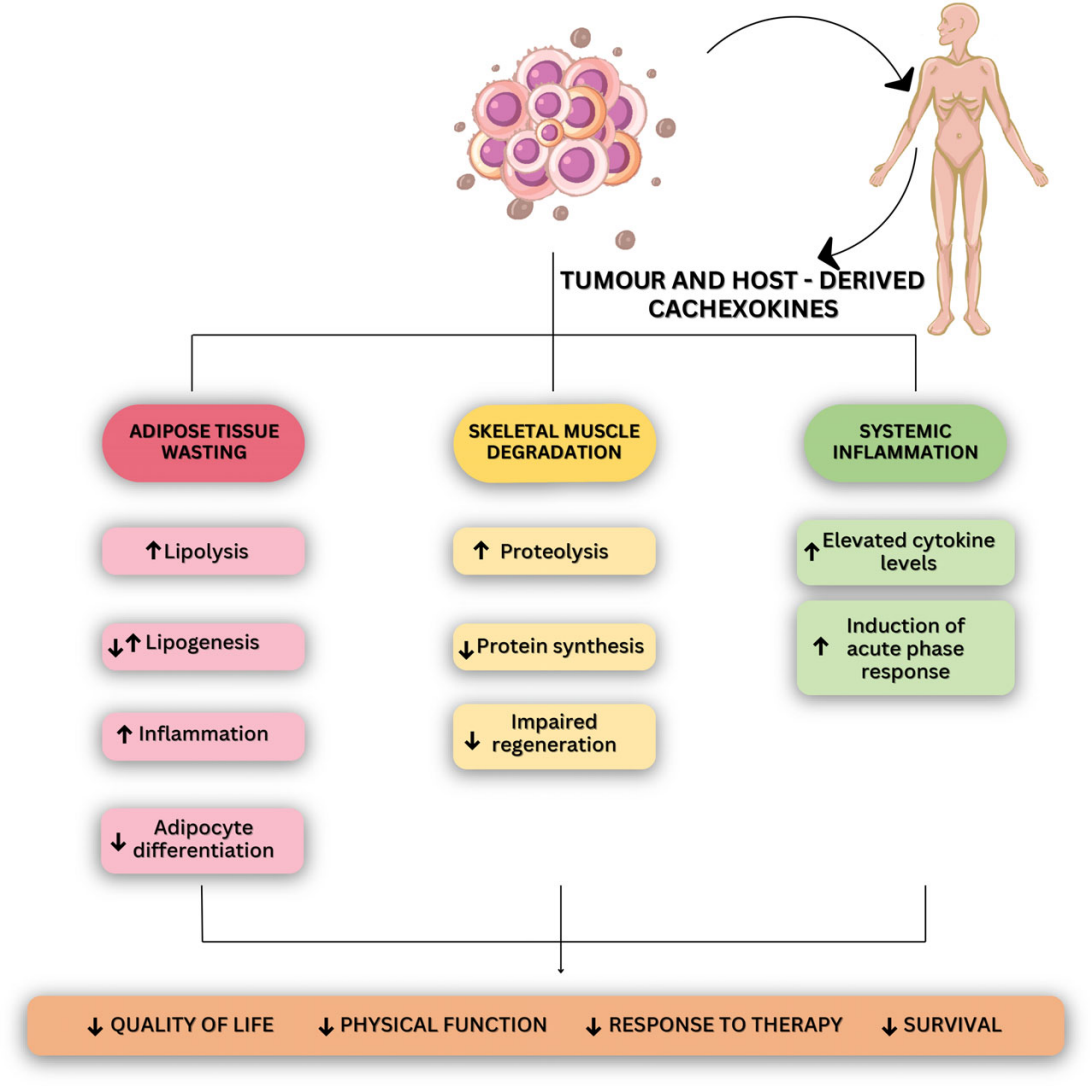
The three characteristics of cancer cachexia highlighted in the current review. Tumour‐ and host‐secreted factors induce adipose tissue wasting, skeletal muscle degradation and systemic inflammation through various molecular mechanisms. The combination of these effects negatively influences several aspects of patient health.

Most cancer patients show both adipose tissue and skeletal muscle wasting (Fig. 2), yet the recent TRACERx study has highlighted different clinical subtypes of Ccx in which adipose tissue loss is not always apparent [19]. When present, adipose tissue loss is one of the earliest events of cachexia, often preceding skeletal muscle loss [15]. It is predominantly driven by a combination of increased lipolysis [15] and altered lipogenesis [2, 20], leading to a functional imbalance of fat storage, lipid loss and a rise in resting energy expenditure [21, 22]. Altered activity and/or levels of key enzymes of lipolysis [adipose triglyceride lipase (ATGL) and hormone sensitive lipase (HSL)] cause increased triglyceride hydrolysis and subsequently elevated levels of circulating free fatty acids and glycerol [15, 23]. Knockout of *Atgl* or *Hsl* in mice counteracts cancer‐induced adipose tissue loss and partially prevents muscle wasting [24]. Lipolysis‐derived metabolites are used within the adipose tissue and in distal organs and can generate energy‐consuming futile cycles, driving for instance gluconeogenesis in the liver or triglyceride re‐esterification in adipocytes. The latter can significantly contribute to overall energy expenditure [25] and may participate in elevated energy wasting in cachectic adipose tissue as evidenced by the strong decline in ATP [2, 26]. Reduced activation of AMP‐activated protein kinase (AMPK) in Ccx—despite normally being activated in states of low cellular energy—may mediate the increased lipolysis/re‐esterification cycling [2]. A formal proof of this cycling is still needed, for instance by tracing of fatty acids within the triglyceride pool using a multilabel multiplex tracing method as recently described [25]. In contrast to the proposed increase in triglyceride re‐esterification, some studies have reported reduced lipogenesis [20, 27, 28], in line with a diminished expression of the lipogenic enzymes fatty acid synthase (FAS), acetyl‐CoA carboxylase (ACC) and diacylglycerol O‐acyltransferase (DGAT) in adipose tissue of cachectic mice. Adipokines may play an important role in regulating lipolysis and lipogenesis in cachexia, as cachectic patients show altered circulating levels of adiponectin [29, 30] and reduced levels of leptin [31]. Changes in circulating leptin levels might not only affect adipose tissue functionality but also immune responses, as leptin regulates immune cell function [32].

Cachectic mice have smaller and more heterogeneous adipocytes, and their mitochondria are more electron dense with an elevated number of cristae compared to weight‐stable control mice [20]. However, uncoupling protein 1 (UCP1)‐dependent browning or brown fat activity do not seem to play a role for cachectic patients as recently evidenced [33, 34]. Especially in the late stages of Ccx, inflammation and fibrosis of adipose tissue occur [20, 35, 36], characterized by increased infiltration of inflammatory cells such as macrophages or T lymphocytes. Key adipogenic factors, such as peroxisome proliferator‐activated receptor gamma (PPARγ) or CCAAT enhancer‐binding protein alpha (CEBPα) are affected by cachexia, influencing not only adipogenesis but also mature adipocyte function by influencing triglyceride synthesis and storage and adipose tissue maintenance [20, 35].

Skeletal muscle loss [15] is one of the most devastating hallmarks of cachexia, being directly linked to about 30% of all cancer‐related deaths [8], with wasting of cardiac, chest and diaphragm muscle often leading to respiratory and cardiac failure [37, 38]. Repression of protein synthesis in combination with elevated protein breakdown mainly mediates the skeletal muscle wasting, orchestrated by the interplay of two major pathways, the ubiquitin proteasome system (UPS) [39] and autophagy [40]. Under physiological conditions, these systems clean up damaged proteins, but in Ccx, their abnormal regulation causes excessive protein degradation. The activation of distinct transcription factors such as nuclear factor κ‐light‐chain‐enhancer of activated B cells (NF‐κB) and forkhead box O (FoxO) induces the two E3 ligases muscle RING finger 1 (MuRF1) and muscle atrophy F box (MAFbx, also termed Atrogin 1), essential drivers of muscle atrophy‐related proteolysis [41]. Both are upregulated in muscle of several Ccx mouse models [13], and inhibition [42] or lack of MuRF1 [43] or Atrogin1 [44] partially prevents mice from wasting. In addition to the UPS, activation of autophagy associates with Ccx in mice [40, 45] and patients [46], and is potentially regulated by FoxO3 activation, controlling the autophagy‐related genes BCL2 interacting protein 3 (Bnip3) and microtubule‐associated protein 1A/1B‐light chain 3 (LC3) [47]. FoxO in turn is regulated by AKT–mTOR, and activation of the AKT–mTOR pathway in muscle reverses muscle wasting [48]. Perturbation of neuromuscular junctions and muscle denervation enhances muscle wasting in cachexia, which is induced by cancer‐induced reductions in bone morphogenetic protein (BMP) [49]. Myonuclear apoptosis triggered by mitochondrial dysfunction further contributes to muscle wasting in Ccx [50], characterized for instance by the presence of apoptotic factors including Caspase 8 and 9 [50], B‐cell leukaemia/lymphoma 2 (BCL2)‐associated X protein (BAX) [51] and DNA fragmentation in cachectic skeletal muscles [52].

Chronic systemic inflammation is a critical component of Ccx, with inflammatory cytokines including interferon gamma (IFNγ) [53], IL‐6 [54, 55], TNFα [56] and IL‐1β [57] being essential drivers of many symptoms of the disease. These tumour‐ or host‐secreted cytokines have prominent effects on several cachexia‐affected organs including the brain, skeletal muscle and adipose tissue. For instance, IL‐6, TNFα and IL‐1 act directly on the brain to enhance anorexia [58, 59]. Intracerebroventricular (ICV) injection of TNFα or IL‐1β at pathophysiological levels—aiming to administer the cytokines directly to the central nervous system—induces anorexia [60, 61]. In the muscle, inflammatory cytokines promote wasting by activating NF‐κB signalling, causing muscle protein breakdown and inhibiting protein synthesis [62, 63]. In adipose tissue, cytokines affect lipolysis and lipogenesis [64, 65, 66], and an IL‐6‐dependent crosstalk between tumour, muscle and fat promotes wasting [55]. IL‐6 also modulates C‐reactive protein (CRP) synthesis, which as part of the acute phase response is mainly produced in the liver via NF‐κB and signal transducer and activator of transcription 3 (STAT3) signalling [67, 68]. High levels of STAT3 associate with Ccx [69, 70]. High CRP is a marker of systemic inflammation and used as diagnostic marker in patients [17, 71, 72].

Additional symptoms of Ccx include anaemia, asthenia, induction of an acute phase response in the liver, dysregulated hormone secretion in the brain and futile energy wasting cycles within and between tissues, recently reviewed for instance in Refs. [9, 22, 73]. Altogether, Ccx‐induced systemic alterations lead to a strong decrease of the patients' quality‐of‐life due to weakness and fatigue, while at the same time limiting therapeutic options as Ccx reduces the responsiveness and tolerance to anti‐cancer therapies, thereby shortening overall survival [7, 74]. To date, only Japanese health authorities have approved anamorelin [75], an appetite enhancer, to counteract anorexia in cachectic patients, whereas the remaining countries do not currently have an approved routine therapy to reverse body wasting in cancer patients.

## 3 Biomarkers in cachexia

Cachexia is a progressive and deteriorating condition that occurs alongside the advancement of cancer, eventually culminating in refractory cachexia. To mitigate the adverse effects of cachexia on anti‐cancer treatment, it is crucial to intervene against its progression at the earliest stages. This underscores the significance of timely identification of its onset through the utilization of clinically validated biomarkers. Indeed, some therapeutic approaches have only shown encouraging results when pre‐cachectic patients with mild weight loss were treated [76]. The combination of weight loss and wasting of muscle mass or strength has been clinically evaluated and established as a marker of Ccx [77]. Indeed, muscle and adipose tissue wasting may occur up to 18 months prior to the clinical diagnosis of cancer [78], serving not only as cachexia but potentially also cancer biomarkers. However, inter‐individual variations such as body composition, initial body mass index, muscle mass, genetic predisposition, physical activity and comorbidities can influence the development of weight and muscle loss, and often delay the diagnosis [6, 79]. Even without obvious loss of total body mass, cancer patients can suffer from ‘hidden cachexia’ [80]. This term refers to weight loss or loss of functional muscle mass that is masked by obesity, large tumours, ascites or oedema. Hence, it is important to support the measurements of muscularity with novel biomarkers that identify cachexia objectively and reliably, independent of weight loss.

**Fig. 3:**
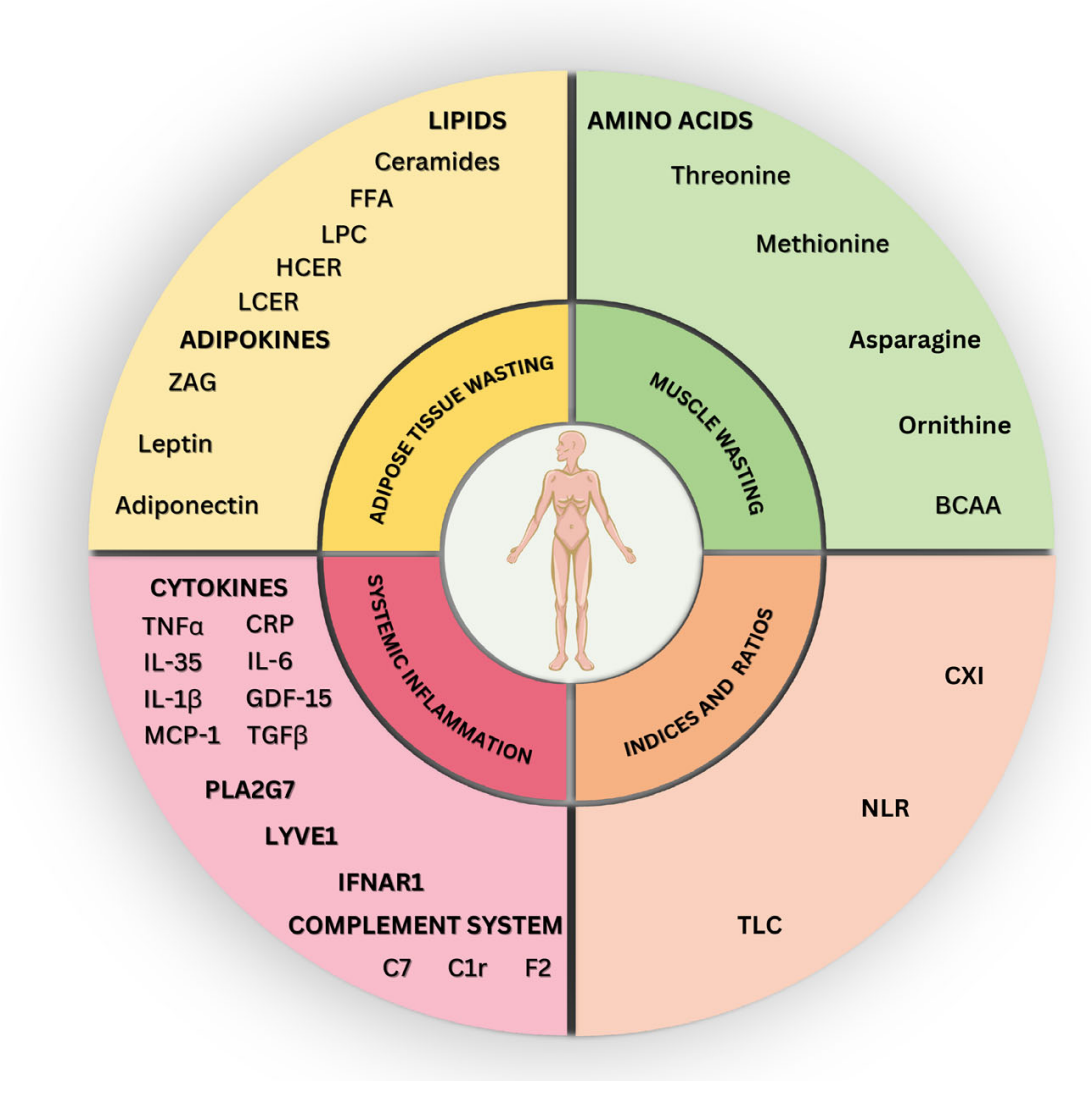
Biomarkers in cancer cachexia. Shown are several cachexia markers, grouped according to the three characteristics of cachexia highlighted in the current review: adipose tissue wasting, skeletal muscle wasting and systemic inflammation. In addition, new indices and scores are shown, aiming to improve biomarker validity by integrating multiple biomarkers. Abbreviations shown in abbreviation list.

Ideal Ccx biomarkers should be detectable in pre‐cachectic patients and exhibit gradual levels during disease progression for effective staging. Preferably, biomarkers should be easily sampled through noninvasive methods such as plasma/serum detection rather than requiring invasive tumour or muscle biopsies, and should allow for straightforward and cost‐effective quantification. Lastly, a valid biomarker should specifically change based on the presence and progression of Ccx without being influenced by other factors like therapy, comorbidities, age or infections. In this chapter, we describe and discuss recent advances in biomarker discovery based on the three aforementioned disease mechanisms of adipose tissue loss, muscle wasting and systemic inflammation (Fig. 3).

### 3.1 Adipose tissue wasting—circulating lipids and adipokines

With lipid metabolism and adipose tissue function being prominently altered in cachexia early on, it is evident that circulating lipids and adipokines will likely be affected by the disease. Indeed, altered circulating adipose tissue‐derived factors and ectopic lipid accumulation have previously been discussed in the context of metabolic disease, as well as cancer [21]. Elevated plasma sphingolipids [3], including ceramides (CERs), hexosyl‐ceramides (HCERs), sphingomyelins (SMs), as well as depleted lysophophatidylcholines (LPCs) [3, 81, 82, 83], and a strongly altered FFA profile are characteristic for Ccx in both mice and men [3, 84]. In line with this, specific lipid species belonging to the aforementioned classes were shown to bear great potential as diagnostic biomarkers in preclinical Ccx models and patients [3]. Moreover, sphingolipids already serve as biomarkers in different cancer types, including ovarian and advanced stage colorectal cancer [85], both prone to induce cachexia. Hence, future studies to evaluate the use of plasma lipids as biomarkers in cachexia seem promising and important.

Adipose tissue remodelling in Ccx also results in altered secretion patterns of several adipokines including zinc‐alpha2‐glycoprotein [86, 87, 88], leptin [89, 90, 91, 92, 93] and adiponectin [29, 30, 94, 95, 96]. However, measurements of circulating levels of these adipokines have led to contradictory results and might relate to the heterogeneous and time‐dependent remodelling of the different adipose tissue depots [97, 98], or differences in sex, BMI or underlying diseases [99, 100, 101, 102] in Ccx. Overall, based on these controversial results, so far, adipokines show low biomarker potential, and further studies are needed to elucidate which parameters influence their regulation in Ccx.

### 3.2 Skeletal muscle degradation—amino acids as biomarkers in Ccx

The marked wasting of lean mass, especially skeletal muscle, via the activation of the UPS and autophagy pathways, results in perturbations of amino acid (AA) metabolism, releasing AAs into the circulation, which can be subsequently metabolized by the tumour or other highly metabolic tissues such as the liver [103]. Indeed, several unbiased metabolomic studies have highlighted changes in AA metabolism as a prominent feature of Ccx in both patients and animal models [81, 82, 104]. These have overall shown that multiple AAs are reduced in the circulation even at early stages of the disease, which may indicate cachectic hypermetabolism. Indeed, in cachectic mice, circulating AAs are among the earliest and most significant metabolic markers, with a decrease of methionine, asparagine and ornithine starting 4–5 days prior to weight loss [104]. In addition, higher plasma branched chain amino acids were detected in patients with pancreatic cancer and were predictive of future muscle loss [105]. A comprehensive review by Ragni *et al*. [106] has recently summarized the role of AAs in cancer metabolism, concluding that AAs might not only be strong Ccx biomarkers but also bear potential in controlling nutritional status of the patient, tumour growth and host microbiota. In addition, investigating the urinary profile of cachectic cancer patients using ^1^H‐NMR (nuclear magnetic resonance) identified the AAs leucine, isoleucine, valine, alanine, threonine, tyrosine, glutamine and serine as characteristic of Ccx [107]. In summary, AAs bear great potential as Ccx biomarkers due to easy sampling in blood or urine and their early changes before symptom onset. Future studies should verify and refine these already present results.

### 3.3 Systemic inflammation—inflammatory proteins as biomarkers in ccx

Systemic inflammation is one of the key drivers of Ccx, with several inflammatory cytokines playing a significant role in disease development. In the last decades, several of these inflammatory markers have been elucidated as biomarkers for Ccx, including TNFα, IL‐6 [79, 108], CRP [69, 70], monocyte chemoattractant protein‐1 (MCP‐1) [109, 110], transforming growth factor‐β (TGFβ) [111] and growth differentiation factor 15 (GDF15) [19, 112, 113]. While inflammatory markers are routinely assessed in cancer patients and clear links to weight loss and survival exist, they are also heavily influenced by additional factors such as infections [114, 115], lifestyle [116] (CRP), anorexia [117], age [13] (GDF‐15) or sexual dimorphism [109, 110] (MCP‐1). In addition, some controversy regarding the translatability from rodent models to humans exists, particularly for TNFα as comprehensively discussed in Refs [79, 108]. As many of the cachexia‐inducing factors that have been assessed so far do not fulfil all characteristics that are required for a diagnostic and therapeutic biomarker, the search for new Ccx markers is still ongoing. In this regard, IL‐35 [118], phospholipase A2 group VII [119], complement C1r subcomponent [120], complement component C7 [121] and interferon alpha and beta receptor subunit 1 [121] have recently been identified, and future studies are needed to investigate their potential in routine settings. In the meantime, new indices and ratios that unite distinct markers, such as cytokines, lipids or AAs, were generated to improve the accuracy of Ccx detection. Amongst these ratios, the neutrophil‐to‐lymphocyte ratio (NLR) is an established clinical marker for systemic inflammation with low costs and easy sampling that has already been associated with Ccx onset [111, 122, 123, 124]. By combining the circulating levels of tissue inhibitor of metalloproteinases‐1 (TIMP‐1)—which has been previously correlated with Ccx in patients—with cachexia‐associated liver parameters (C‐reactive protein, ferritin, albumin, total protein and gamma‐glutamyl transferase), the TIMP1/liver/cachexia (TLC) score shows promising potential to detect Ccx [125]. The Cachexia Index (CXI) combines markers for systemic inflammation (NLR), nutritional status (serum albumin) and muscle wasting (skeletal muscle index) [126], and can be independently applied in men and women [126]. Circulating cytokine levels are currently used as a diagnostic factor for cachexia according to some definitions (Evans [17], cachexia score (CASCO) [127], Glasgow score [71]). Yet the current difficulties in defining universal factors and thresholds underline the importance of incorporating confounding factors such as BMI, metabolic health, nutrition, infections and age into the equation [128, 129, 130]. How these confounders affect diagnostic factors should thus be investigated more thoroughly.

Overall, biomarker research has strongly progressed in the last decade and has identified novel circulating protein and non‐protein markers for Ccx. Moreover, new indices and ratios were established, and future studies taking tumour entities, sex differences and ageing into account will test their validity.

## 4 The underestimated effect of age on cachexia

**Fig. 4:**
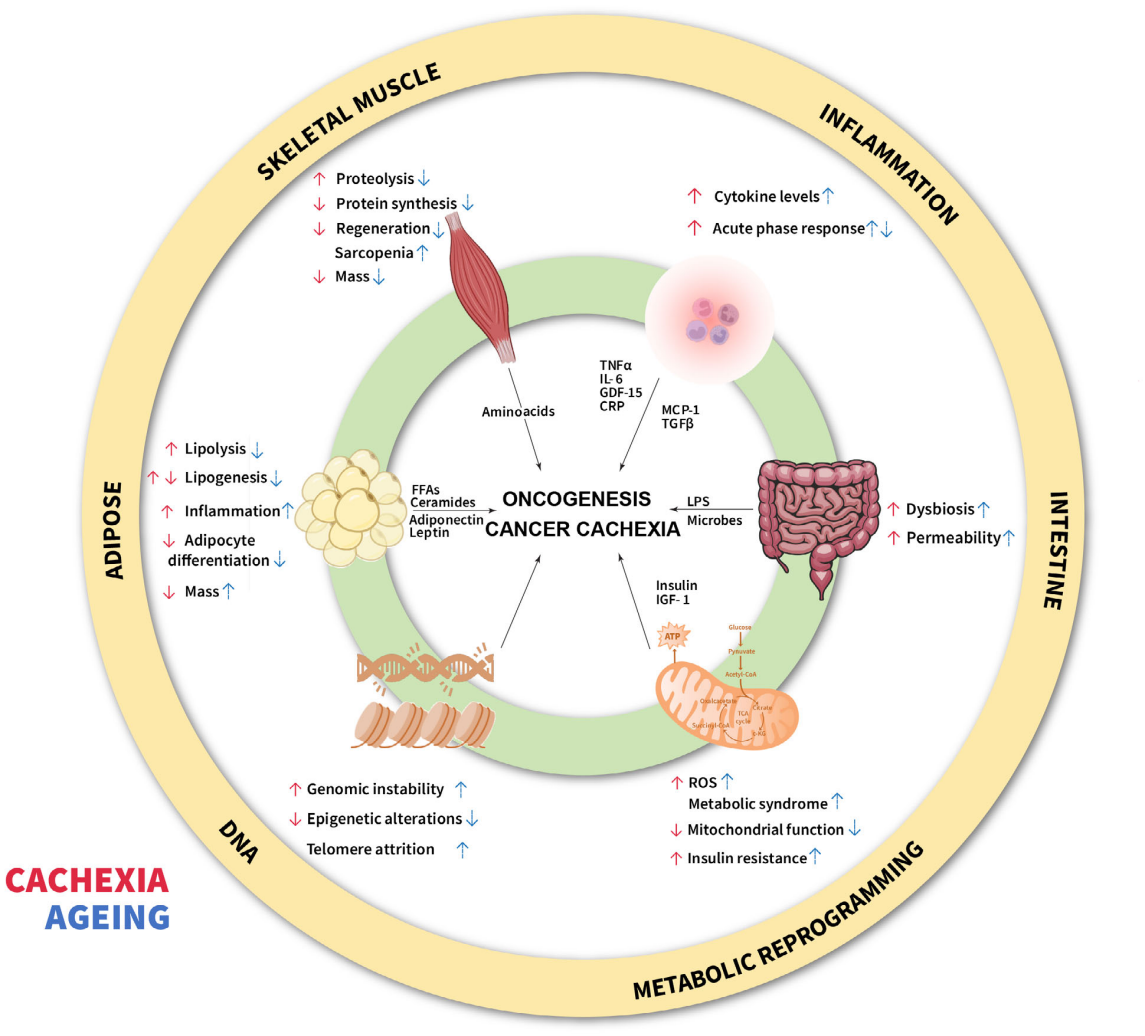
Common pathways and circulating factors in cachexia and ageing. The aged population shows an increased risk of developing cancer and its accompanying syndrome cancer cachexia. Metabolic, genomic and behavioural alterations upon ageing increase the risk for cancer and Ccx development. Red arrows indicate changes in cachexia, blue/dotted arrows indicate changes in ageing. Abbreviations shown in abbreviation list.

Ageing was recently shown to affect both the validity of Ccx biomarkers and Ccx development in mice [13]. As the world population is ageing, cancer incidence and as a result cachexia incidence will likely rise in the future [131]. Indeed, the global cancer burden was estimated to increase by 47% in 2040 (28.4 million new cases) compared to 2020 (19.3 million cases) [132], and while the risk of developing cancer before the age of 40 is 2%, it increases tremendously to about 50% by the age of 80 [133]. As the age‐related increase in cancer risk is well accepted, many researchers have contributed to deciphering the rather complex mechanisms of how ageing influences cancer onset [133, 134, 135] (Fig. 4). In 2023, López‐Otín *et al*. have combined the well‐known hallmarks of cancer [136] with their previously published hallmarks of ageing [137] to establish meta‐hallmarks that show very close parallels between cancer and ageing [138]. Among those, genomic instability, epigenetic alterations, chronic inflammation and dysbiosis associated with oncogenesis. In addition, metabolic reprogramming is a well‐established hallmark of both cancer [139] and ageing [140]. Metabolic dysfunction in ageing involves chronic dysregulation of cellular and host bioenergetic programs, leading to changes in cellular function and an increased susceptibility to metabolic disorders. In line with this, the metabolic syndrome occurs increasingly with age and is associated with obesity, dyslipidaemia, hyperglycaemia, insulin resistance and cardiovascular diseases [141]. Furthermore, ageing is frequently associated with age‐related loss of muscle mass and function, sarcopenia. In the next section, we discuss possible interactions between ageing and Ccx in the context of adipose tissue and muscle dysfunction and inflammation.

### 4.1 Adipose tissue dysfunction

In addition to the metabolic alterations during ageing, body composition changes in the elderly. Ageing results in visceral adiposity, in contrast to Ccx. However, similar to Ccx, adipose tissue is dysfunctional in aged individuals, promoting low‐grade chronic inflammation and insulin resistance [142]. Hence, ageing results in increased adipocyte expression of pro‐inflammatory cytokines such as IL‐6 or TNFα [143]. In contrast to Ccx, catecholamine‐induced lipolysis declined by 50% upon ageing [144], which may be mediated by adipose tissue macrophages lowering the bioavailability of noradrenaline [145]. In humans, mature adipocytes appear to be the main modulators of age‐induced alterations in catecholamine‐induced lipolysis [146, 147]. Infiltrating B cells further modulate adipose tissue lipolysis in aged mice, and systemic B‐cell depletion restored levels of ATGL and HSL [148]. Ageing also reduced adipose tissue expression of MGL [147], FASN and DGAT2 [149], indicative of reduced lipogenesis. Hence, contrary to Ccx, both decreased lipolysis and lipogenesis are characteristics of age‐related adipose tissue dysfunction, which may reduce the tissue's metabolic flexibility, i.e. alter its ability to undergo cachexia‐related metabolic adaptations. Chronic inflammation and immune cell infiltration seem to play key roles in mediating adipose tissue dysfunction during ageing. Consequently, age also affects the secretion of adipokines, as increased adiposity in the elderly leads to elevated circulating leptin levels. However, with a similar BMI, plasma leptin levels were lower in aged compared to young individuals, especially in women [150, 151]. Circulating adiponectin levels correlated positively with age in both sexes [151]. Also, ceramides increased in aged individuals [152]. Overall, age‐related changes in white adipose tissue mass, functionality and inflammation might promote Ccx pathogenesis.

### 4.2 Muscle dysfunction

One of the most prominent body composition changes upon ageing is a decrease in lean mass, also termed sarcopenia [153, 154]. Importantly, the term sarcopenia is specifically linked to loss of muscle mass and strength in ageing and thereby discriminates muscle loss due to ageing from other causes such as cancer‐induced wasting [155]. The influence of age on cachexia and the interplay between sarcopenia and cachexia have not been addressed in detail so far. Being increasingly recognized as geriatric syndrome, by the age of 80, individuals have approximately lost 30% of their muscle mass [156], leading to elevated risk of functional impairment, disability and mortality [157]. Muscle changes in age‐associated sarcopenia are manifold and substantial [158]. Briefly, decreased protein synthesis and low protein and caloric intake contribute to sarcopenia development, as do genetic risk factors, neurodegenerative processes and muscle fibre atrophy [155]. Aged muscle shows impaired anabolic flexibility [159] and reduced protein synthesis rates linked to increased inflammation [160]. Reduced muscle insulin sensitivity during ageing reduces the anabolic actions of the Akt/ mTOR signalling pathway. Furthermore, impaired mitochondrial function of aged muscle compromises energy supply and induces oxidative stress, which affects multiple downstream signalling pathways including activation of JNK signalling, AMPK signalling and endoplasmic reticulum stress, all important for maintaining muscle mass [161]. Decreased lysosomal function and autophagy in age contribute to muscle wasting and reduced muscle innervation in sarcopenia as damaged or misfolded proteins accumulate [162]. Whether the Ccx‐regulated atrogenes MuRF1 and Atrogin1 play a role in age‐related sarcopenia is somewhat debated, with a majority of reports stating that they are not regulated by sarcopenia [163, 164, 165].

In line with the decrease of lean mass, aged individuals with sarcopenia display a distinct AA profile [166] with lower levels of methionine, an essential AA that also decreased in cachectic mice, even before weight loss [104, 166]. These metabolic changes might further foster the development of Ccx, given that changes in circulating AA levels are early predictors for cachexia as described in more detail above [104]. Overall, sarcopenia and Ccx may be additive and age‐related loss of muscle mass and function may further contribute to the fast functional decline seen in patients with cancer.

### 4.3 Inflammation

Chronic systemic inflammation represents another hallmark of ageing, termed inflammageing [140]. This chronic low‐grade inflammation in the elderly comes along with slight increases of circulating adipokines, chemokines and proinflammatory cytokines, such as IL‐6 [167] and TNFα [168]—even in the absence of a clinically active disease [169]. It contributes to various pathologies including cancer [170], cardiovascular disease [171] and sarcopenia [172, 173]. Not only genetic susceptibility and visceral obesity but also alterations in gut permeability and cellular senescence might drive inflammageing, as comprehensively reviewed by Ferrucci *et al*. [174]. In line with these findings, inflammageing has been linked to a higher mortality [179, 180]. Many circulating factors related to Ccx‐induced inflammation are also elevated in ageing, such as CRP [175], GDF‐15 [176], MCP‐1 [177], TGFβ [178], TNFα [168] and IL‐6 [167]. The age‐related upregulation of these factors might ease the development of Ccx.

Based on all aforementioned mechanistic and metabolic changes that we face with increasing age, it is crucial to pay more attention to the elderly when designing studies, as age strongly influences outcomes [13]. In cancer research, scientists have already started to address the effect of ageing on treatment and therapy success [181]. However, studies about the influence of ageing on cachexia pathogenesis are still scarce to date, despite metabolic dysregulation, skeletal muscle loss, reduced food intake and systemic inflammation being strong hallmarks of both ageing and Ccx, hence potentially making elderly individuals particularly vulnerable to cachexia. Indeed, cachexia is more prevalent in cancers of older age, as for example gastric cancer [182]. Additional factors such as metabolic dysfunction associated with diabetes might further impact cachexia development [12] in addition to ageing.

Ruan *et al*. [183] have reported a new index to estimate prognosis, overall survival and malnutrition in cancer patients, termed the Geriatric Nutritional Risk Index (GNRI). The GNRI is based on the patient's serum albumin levels and bodyweight, with a low GNRI being associated with worse prognosis, lower overall survival and malnutrition. When compared to non‐cachectic cancer patients, cachectic patients displayed a significantly lower GNRI. Additionally, aged cancer patients (> 70 years) had a markedly reduced GNRI compared to patients younger than 70 years, indicating elevated mortality risk [183]. Hence, performing cachexia screenings early in elderly cancer patients is important to counteract Ccx at the earliest time point possible, thereby improving disease outcome. Takeda *et al*. [184] have investigated how Ccx and sarcopenia influenced the treatment success in pancreatic cancer patients receiving chemotherapy. Therein, Ccx was associated with higher age, increased inflammatory markers, an elevated NLR, worse nutritional status, reduced progression‐free survival and early treatment discontinuation, while sarcopenia had less of an impact on the clinical parameters in aged patients with pancreatic cancer [184]. However, this study only investigated an aged patient group and did not include any young patients [184]. While these exemplary studies already emphasize that patient age is of high importance with respect to treatment, research comparing aged and young cachectic cancer patients is still underrepresented. As a first step to adapt cachexia screenings to the ageing population, in 2011 the European Society of Clinical Nutrition and Metabolism (ESPEN) suggested a higher BMI cut‐off value of 22 kg·m^−2^ in older individuals compared to 20 kg·m^−2^ in younger ones [185]. Of note, the impact of ageing should not only be investigated in cancer‐associated cachexia, but also other cachexia‐inducing diseases such as the acquired immunodeficiency syndrome [186] or cardiac cachexia [187] to estimate if ageing further drives cachexia pathogenesis in this context.

Preclinical studies investigating the influence of age on Ccx are still underrepresented. To address this, we have previously assessed Ccx pathogenesis in different frequently used cachexia mouse models of different age groups [13]. We have shown that age had a strong impact on cachexia pathogenesis, which depended on the mouse line and strain. While the C26 model, using BALB/c mice, was unaffected by age (as also previously reported by Talbert *et al*. [188]), we found that LLC‐implantation into aged C57BL/6J mice aggravated cachexia. Furthermore, while LLC cells did not induce cachexia in young C57BL/6N mice, they did induce wasting in aged C57BL/6N mice. These data highlight the importance of validating results in more than one preclinical model and ideally additionally in an aged mouse cohort. With respect to Ccx biomarkers, ageing affected the validity of the currently used markers IL‐6 and IL‐1β in both mice and patients [13]. While young patients with an age ≤ 55 years showed significant correlations of the aforementioned biomarkers with bodyweight loss, this significance was lost upon ageing (> 55 years), highlighting the strong impact of age that should be taken into account when examining cancer patients and cachexia onset, even based on already established biomarkers [13]. In the future, systematic studies investigating cachexia development and biomarker consistency in dependence of age, considering specific metabolic vulnerabilities of different age groups, will be necessary to streamline optimal diagnosis and treatment options to patients' needs.

## Author contributions

JG and MR conceptualized the review. JG wrote the first version of the manuscript. MR edited the manuscript and co‐wrote the final version.

## Conflict of interest

The authors declare no conflict of interest.
